# Mitochondrial ATP concentration decreases immediately after glucose administration to glucose‐deprived hepatocytes

**DOI:** 10.1002/2211-5463.13744

**Published:** 2023-12-13

**Authors:** Saki Tsuno, Kazuki Harada, Mina Horikoshi, Marie Mita, Tetsuya Kitaguchi, Masami Yokota Hirai, Mitsuharu Matsumoto, Takashi Tsuboi

**Affiliations:** ^1^ Department of Life Sciences, Graduate School of Arts and Sciences The University of Tokyo Tokyo Japan; ^2^ Dairy Science and Technology Institute Kyodo Milk Industry Co., Ltd. Tokyo Japan; ^3^ Department of Biological Sciences, Graduate School of Science The University of Tokyo Tokyo Japan; ^4^ Laboratory for Chemistry and Life Science, Institute of Innovative Research Tokyo Institute of Technology Yokohama Japan; ^5^ RIKEN Center for Sustainable Resource Science Yokohama Japan; ^6^ Present address: Biomedical Research Institute National Institute of Advanced Industrial Science and Technology Osaka Japan

**Keywords:** ATP, glucose‐deprivation, hepatocyte, live‐cell imaging, mitochondria

## Abstract

Hepatocytes can switch their metabolic processes in response to nutrient availability. However, the dynamics of metabolites (such as lactate, pyruvate, and ATP) in hepatocytes during the metabolic switch remain unknown. In this study, we visualized metabolite dynamics in primary cultured hepatocytes during recovery from glucose‐deprivation. We observed a decrease in the mitochondrial ATP concentration when glucose was administered to hepatocytes under glucose‐deprivation conditions. In contrast, there was slight change in the cytoplasmic ATP concentration. A decrease in mitochondrial ATP concentration was associated with increased protein synthesis rather than glycogen synthesis, activation of urea cycle, and production of reactive oxygen species. These results suggest that mitochondrial ATP is important in switching metabolic processes in the hepatocytes.

## Graphical abstract

Hepatocytes can switch their metabolic processes in response to nutrient availability. We visualized metabolite dynamics in primary cultured hepatocytes during recovery from glucose‐deprivation. Glucose administration to hepatocytes under glucose deprivation conditions can cause an immediate decrease in the mitochondrial ATP concentration. In contrast, cytoplasmic ATP concentration showed little change.

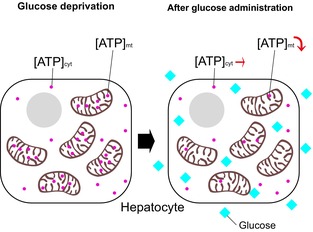

**Abbreviations**
AMPK: AMP‐activated protein kinase
AUC: area under the curve
BSA: bovine serum albumin
DMEM: Dulbecco's modified Eagle's medium
4E‐BP1: eukaryotic translation initiation factor 4E‐binding protein 1
FBS: fetal bovine serum
FI: fluorescence intensity
HBSS: Hanks' balanced salt solution
HPLC: high‐performance liquid chromatography
LI: luminescence intensity
mTORC1: mammalian target of rapamycin complex 1
NAFLD: non‐alcoholic fatty liver disease
NASH: non‐alcoholic steatohepatitis
OCR: oxygen consumption rate
OXPHOS: oxidative phosphorylation
P/S: penicillin–streptomycin
RB: Ringer's buffer
ROS: reactive oxygen species

The liver is a critical hub for numerous physiological processes that support metabolism, immunity, digestion, detoxification, vitamin storage, and other functions. Among these functions, appropriate provision and retrieval of energy sources, particularly glucose, is essential for systemic energy homeostasis [1]. Blood glucose levels decrease during fasting, and the liver supplies glucose to extrahepatic tissues via glycogenolysis and glyconeogenesis. Upon feeding, sufficient glucose from dietary sources becomes available and fasting‐induced responses disappear quickly [2]. During these environmental changes, hepatocytes, which are the major components of the liver, are capable of switching metabolic processes. Multiple pathways regulate energy metabolism in hepatocytes at the molecular level [3, 4]. Abnormalities in these signaling pathways have been implicated in nonalcoholic fatty liver disease (NAFLD) and/or type 2 diabetes [1].

Changes in gene expression and autophagy have been increasingly reported as key factors in the energy dynamics of hepatocytes during fasting and feeding [5, 6, 7]. A majority of analyses have been performed on liver samples taken several hours after refeeding [8, 9, 10], and there are few reports of responses immediately after refeeding. The regulation of intracellular metabolite levels (lactate, pyruvate, and ATP) in hepatocytes may play a role in the quick response to refeeding; however, the dynamics of these metabolites in hepatocytes during refeeding after fasting are not clear.

Here, we investigated the molecular basis of immediate responses to refeeding. We visualized the dynamics of metabolites in mouse primary hepatocytes using fluorescent protein‐based indicators, maintained the hepatocytes under glucose‐deprivation conditions, and observed the responses of intracellular glucose, ATP, pyruvate, and lactate after administration of high concentration of glucose, through which we simulated the conditions of fasting and refeeding in animals. We found that in glucose‐deprived hepatocytes, mitochondrial ATP concentration decreased immediately after glucose administration. Our results provide insights into the effect of nutrient repletion after a state of depletion on the metabolic status of hepatocytes and may help elucidate the potential of dietary interventions in living organisms.

## Materials and methods

### Chemicals

Guaiacol and rapamycin were purchased from Tokyo Chemical Industry (Tokyo, Japan). Dimethyl sulfoxide was purchased from FUJIFILM WAKO Pure Chemical Industries (Osaka, Japan).

### Ethics statement

All experiments involving mice were approved by the Animal Experiment Ethics Committee of the Graduate School of Arts and Sciences at the University of Tokyo (approval no. 2021‐3). The experiments were conducted in accordance with the guidelines of the Life Science Research Ethics and Safety Committee of the University of Tokyo.

### Animals

Male ICR mice (purchased from Sankyo Labo Service Corporation, Tokyo, Japan) were used to avoid the possible effects related to estrous cycles. The mice were housed under temperature‐ (24 ± 2 °C) and humidity‐controlled (55 ± 10%) conditions, with a 12‐h light/dark cycle and *ad libitum* access to food and water throughout the study. To establish a healthy mouse model, mice were raised on a standard chow diet (CE‐2; CLEA Japan, Tokyo, Japan) and used for primary culture at 7–9 weeks of age. For the obese model, mice were fed a commercially available high‐fat diet (Research Diets 12492; 20% protein, 20% carbohydrate, and 60% fat 5.24 kcal·g^−1^) from 3 to 9 weeks of age.

### Isolation of primary hepatocytes

After 7‐ to 9‐week‐old mice were anesthetized with isoflurane, a 27‐gauge needle connected to a tube and a perfusion pump was inserted into the portal vein, and the inferior vena cava was cut to allow blood to flow out. The liver was washed with prewarmed Hanks' balanced salt solution (HBSS without Ca^2+^, Mg^2+^, and phenol red; #17461‐05, Nacalai Tesque, Kyoto, Japan) supplemented with 25 mm HEPES (#17557‐94, Nacalai Tesque) and 0.5 mm EDTA. The cells were then perfused with HBSS (containing Ca^2+^, Mg^2+^, and phenol red #17459‐55, Nacalai Tesque) supplemented with 25 mm HEPES and 1 mg·mL^−1^ Liberase (Sigma‐Aldrich, St. Louis, MO, USA). After mechanical dispersion, the cells were passed through a 70 μm cell strainer and centrifuged at 50 ***g*** for 5 min. The pelleted hepatocytes were gently resuspended in Dulbecco's modified Eagle's medium (DMEM) containing 5 mm glucose, 10% fetal bovine serum (FBS: Thermo Fisher Scientific, Waltham, MA, USA), and 1% penicillin–streptomycin (P/S; Nacalai Tesque) (low‐glucose medium). An equivalent isotonic Percoll solution (1:9 ratio of 10× PBS and Percoll Plus, Cytiva, Tokyo, Japan) was then centrifuged at 200 ***g*** for 10 min. The pellet was washed with a low‐glucose medium and centrifuged at 50 ***g*** for 5 min. The final pellet was resuspended in a low‐glucose medium. The cells were seeded in dishes or plates coated with Cellmatrix Type I‐C (Nitta Gelatin, Osaka, Japan). After 3 h, the medium was changed to William's E medium (Sigma‐Aldrich) containing 11 mm glucose, 2 mm glutamine, and 1% P/S and incubated at 37 °C with 5% CO_2_. Two days after seeding, hepatocytes were cultured overnight in low‐glucose medium. On the day of the experiments, the medium was changed to modified Ringer's buffer (RB; 140 mm NaCl, 3.5 mm KCl, 0.5 mm NaH_2_PO_4_, 0.5 mm MgSO_4_, 1.5 mm CaCl_2_, 10 mm HEPES, 2 mm NaHCO_3_) with or without 11 mm glucose to establish a cell model simulating mouse steady state (glucose‐repletion condition) or fasting conditions (glucose‐deprivation condition). A cell model simulating mouse refeeding was established by replacing the medium with RB containing 25 mm glucose.

### Transfection

Hepatocytes (5 × 10^4^ cells) were plated into 40‐mm glass‐bottomed dishes and cultured overnight. The expression plasmids (1.5 μg each) Red Glifon 300, MaLionR, mito‐MaLionR, Green Pegassos, and GEM‐IL3.0 (gift from Masayuki Yazawa) [11, 12, 13, 14] were transfected into hepatocytes using 3 μL of Lipofectamine 2000 transfection reagent (Thermo Fisher Scientific) in 1 mL of William's E medium supplemented with 2 mM glutamine. The cells were incubated at 37 °C in a 5% CO_2_ incubator for 4 h. The medium was changed to William's E medium supplemented with 2 mM glutamine and 1% P/S.

### Live cell imaging

The experimental procedures were mainly based on our previous study [15]. Briefly, single‐color imaging of hepatocytes was performed using an inverted fluorescence microscope (IX‐71, Olympus, Tokyo, Japan) equipped with an oil immersion objective lens (UApo/340, ×40, NA = 1.35, Olympus), EM‐CCD camera (Evolve, Photometrics, Tucson, AZ, USA), and xenon lamp. Images were acquired with a filter set (U‐MWIY2, Olympus) consisting of a 545–580 nm excitation filter, 610 nm emission filter, and 600 nm dichroic mirror for Red Glifon 300, MaLionR, and mito‐MaLionR. For Green Pegassos, a filter set (U‐MWIBA2, Olympus) was used with a 460–495 nm excitation filter, 510–550 nm emission filter, and 505 nm dichroic mirror. For GEM‐IL3.0, a filter set (U‐MWU2, Olympus) with a 330–385 nm excitation filter, 420 nm emission filter, and 400 nm dichroic mirror were used. The objective lens, stage, and perfusion tube were heated to 37 °C. Images were acquired every 5 s using the metamorph software (Molecular Devices, Sunnyvale, CA, USA).

### Measurement of oxygen consumption

Oxygen consumption rate (OCR) was measured using the Extracellular OCR Plate Assay Kit (Dojindo, Kumamoto, Japan) according to the manufacturer's protocol. Briefly, hepatocytes (5 × 10^4^ cells) were plated in each well of a 96‐well black plate. The cells were treated with oxygen probe in RB without or with 11 mm glucose for 30 min at 37 °C. The cells were stimulated with glucose at a final concentration of 25 mm, and mineral oil was added dropwise. The fluorescence intensity was recorded (excitation and emission wavelengths of 500 and 650 nm, respectively) at 10‐min intervals using a microplate reader Varioscan Lux (Thermo Fisher Scientific). The OCR was calculated by analyzing the kinetic profiles obtained from the measurements.

### Measurement of glycolytic/mitochondrial ATP and ADP levels

Total and glycolytic ATP were determined using a glycolysis/OXPHOS assay kit (Dojindo). The ADP levels were measured using an ADP/ATP ratio assay kit‐luminescence (Dojindo). In brief, hepatocytes (6 × 10^3^ cells) were plated in each well of a 96‐well white plate. To measure total and glycolytic ATP, the cells were treated with or without 1.25 μm oligomycin for 1 h. Mitochondrial ATP was calculated by subtracting glycolytic ATP from total ATP. Luminescence was measured using a microplate reader Varioscan Lux (Thermo Fisher Scientific).

### Measurement of intracellular glycogen

Glycogen levels in hepatocytes were determined using the EnzyChrom Glycogen assay kit (BioAssay Systems, Hayward, CA, USA) according to the manufacturer's protocol. Briefly, hepatocytes (2 × 10^5^ cells) plated in each well of 12‐well plates were washed three times in ice‐cold PBS and homogenized by ultrasonication (amplitude 20; 20 s) in 0.025 m citrate buffer (pH 4.2), containing 2.5 g·L^−1^ NaF, on ice. After centrifugation at 14 000 ***g*** for 5 min at 4 °C to pellet the cellular debris, the supernatants were analyzed according to the manufacturer's instructions.

### Measurement of intracellular urea

Urea levels in hepatocytes were determined using the Urea Assay Kit STA‐382 (Cell Biolabs, San Diego, CA, USA) according to the manufacturer's protocol. Briefly, hepatocytes (2 × 10^6^ cells) were plated in a 60‐mm dish. The cells were washed three times in ice‐cold PBS. 200 μL of assay buffer was added to the cells, and incubated for 5 min on ice. The cells were stripped off with a cell scraper, and the cell suspensions were homogenized using a syringe and needle. After centrifugation at 14 000 ***g*** for 10 min at 4 °C to pellet the cellular debris, the supernatants were analyzed according to the manufacturer's instructions.

### Measurement of intracellular l‐arginine

The concentration of l‐arginine in hepatocytes was quantified using high‐performance liquid chromatography (HPLC). Briefly, hepatocytes (1 × 10^6^ cells) were plated in 6‐well plates. The cells were washed three times with ice‐cold PBS. 1 mL of ice‐cold PBS was added to the cells. The cells were stripped off with a cell scraper. The cell suspensions were centrifuged at 1000 ***g*** for 10 min at 4 °C, and the supernatants were removed. The pellets were lysed by pipetting and vortexing with 15 μL of 10% trichloroacetic acid and incubated for 10 min on ice. After centrifugation at 20 400 ***g*** for 10 min at 4 °C, the supernatants were used for derivatization. 10 μL of the supernatants was mixed with 20 μL of 1.5 mL·mL^−1^ 6‐aminoquinolyl‐*N*‐hydroxysuccinimidyl carbamate (Beta Pharma (Shanghai) Co., Ltd., Shanghai, China) in acetonitrile and 70 μL of borate buffer adjusted to pH 8.8 with phosphoric acid. The mixtures were incubated for 10 min at 55 °C. After centrifugation (20 400 ***g*** for 5 min at 4 °C), the supernatants were filtered through a 0.20 μm PTFE syringe driven filter unit (Millex‐LG, Merck Millipore, Darmstadt, Germany) immediately before HPLC analysis. High‐performance liquid chromatography separations were conducted on a Waters Alliance e2695 system (Waters Co., Ltd., Milford, CT, USA) equipped with an autosampler, a temperature‐controlled column, and Waters 2475 multi‐wavelength fluorescence detector. Chromatographic separations were performed on a Waters AccQ.Tag amino acid analysis column (3.9 × 150 mm, 4 μm), equipped with a Nova‐Pak™ C_18_ Sentry™ Guard column (3.9 × 20 mm, 4 μm) (Waters Co., Ltd.). The column was thermostatically controlled at 39 °C. The injection volume was 5 μL. Mobile phases A to D consisted of 100 mm sodium acetate and 5.6 mm triethylamine, adjusted to pH 5.7 with phosphoric acid, 100 mm sodium acetate, and 5.6 mm triethylamine, adjusted to pH 6.8 with phosphoric acid, 100% acetonitrile, and Milli‐Q water, respectively. The gradient conditions are listed in Table 1. Elution was monitored at an excitation wavelength of 250 nm and an emission wavelength of 395 nm. The identification of l‐arginine was performed using the retention time (standard; 28.5 min). The quantification of l‐arginine was performed using an external standard method, and the concentration is expressed as μm.

**Table 1:** Selected gradient elution.

Time (min)	Flow rate (mL·min^−1^)	% A	% B	% C	% D
0.0	1.0	90.0	10.0	0.0	0.0
0.5	1.0	89.0	10.0	1.0	0.0
17.0	1.0	88.0	10.0	2.0	0.0
24.0	1.0	86.0	9.0	5.0	0.0
32.0	1.0	63.0	25.0	12.0	0.0
33.5	1.0	0.0	87.5	12.5	0.0
33.8	1.3	0.0	87.5	12.5	0.0
37.0	1.3	0.0	87.0	13.0	0.0
48.0	1.3	0.0	85.0	15.0	0.0
48.1	1.3	0.0	0.0	60.0	40.0
51.0	1.0	90.0	10.0	0.0	0.0
60.0	1.0	90.0	10.0	0.0	0.0

### Measurement of reactive oxygen species (ROS)

The intracellular ROS levels were assessed using the ROS Assay Kit (Dojindo Laboratory). Briefly, hepatocytes (5 × 10^4^ cells) were plated into each well of a 96‐well black plate. The cells were incubated with working solution (2′,7′‐dichlorodihydrofluorescein diacetate; DCFH‐DA Dye working solution) for 30 min at. The cells were then washed with RB, and the fluorescence intensity was measured (excitation and emission wavelengths of 488 and 520 nm, respectively) using a microplate reader Varioscan Lux (Thermo Fisher Scientific).

### Cell viability test

Viability of hepatocytes was assessed using the Cell Counting Kit‐8 assay (Dojindo Laboratory). Briefly, hepatocytes (2.5 × 10^4^ cells) were plated in each well of a 96‐well plate. The medium was changed to 80 μL of RB with or without 11 mm glucose and 10 μL of the cell counting kit solution. The cells were stimulated with glucose at a final concentration of 25 mm. After 1 h of the addition of the cell counting kit solution, the absorbance (Abs_450_) was measured using a microplate reader Varioscan Lux (Thermo Fisher Scientific).

### Quantitative RT‐PCR

Total RNA was extracted from hepatocytes (1 × 10^6^ cells for *Ckmt1* and 2 × 10^5^ cells for the others) using NucleoSpin RNA (TaKaRa, Shiga, Japan) according to the manufacturer's protocol. cDNA was synthesized by reverse transcription of total RNA using the PrimeScript RT Reagent Kit (TaKaRa). The StepOnePlus real‐time PCR system (Thermo Fisher Scientific) and TB Green Premix Ex Taq II (Tli RNaseH Plus) (TaKaRa) were used for quantitative RT‐PCR of the synthesized cDNA. The 18S rRNA reference genes were used to calculate relative expression levels using the ΔΔ*C*_t_ method. For quantitative RT‐PCR (qPCR), the following primer sequences were utilized: glycogen synthase 2 (*Gys2*, NM_001411624.1) [16], forward, 5′‐ACCAAGGCCAAAACGACAG‐3′ and reverse, 5′‐GGGCTCACATTGTTCTACTTGA‐3′; solute carrier family 2 (facilitated glucose transporter), member 1 (*Slc2a1 (Glut1)*, NM_001424864.1) [17], forward, 5′‐GCAGTTCGGCTATAACACTGG‐3′ and reverse, 5′‐GCGGTGGTTCCATGTTTGATTG‐3′; solute carrier family 2 (facilitated glucose transporter), member 2 (*Slc2a2 (Glut2)*, NM_031197.2) [17], forward, 5′‐TTCCAGTTCGGCTATGACATCG‐3′ and reverse, 5′‐CTGGTGTGACTGTAAGTGGGG‐3′; creatine kinase, mitochondrial 1, ubiquitous (*Ckmt1*, NM_001355069.1) [18], forward, 5′‐TGTCTTCAAGAGTCAGAACTGGC‐3′ and reverse, 5′‐AGCATCCACCACAACACGTT‐3′; Rn18s 18S ribosomal RNA (18S rRNA, NR_003278.3) [19] forward, 5′‐GGACCAGAGCGAAAGCATTTG‐3′ and reverse, 5′‐TTGCCAGTCGGCATCGTTTAT‐3′).

### Western blotting

Total protein was extracted from hepatocytes (2 × 10^5^ cells) with RIPA buffer (Thermo Fisher Scientific) supplemented with a protease inhibitor cocktail (Thermo Fisher Scientific) and a phosphatase inhibitor (Nacalai Tesque). The solution was then centrifuged at 14 000 ***g*** for 5 min at 4 °C to remove the cell debris. The clear supernatant was transferred to a clean tube as a protein extract and stored at −80 °C until further analyses.

The proteins were denatured at 95 °C for 5 min. 8 μg of proteins was separated using a Mini Trans‐Blot Cell (Bio‐Rad, Hercules, CA, USA) system on 4–20% Mini‐PROTEAN TGX Gels (Bio‐Rad). The proteins were transferred onto PVDF membranes using the Trans‐Blot Turbo Transfer System (Bio‐Rad) with preassembled Trans‐Blot Turbo Mini 0.2 μm PVDF Transfer Packs (Bio‐Rad). The membranes were blocked with 3% bovine serum albumin (BSA) for 1 h. Subsequently, the membranes were incubated with primary antibodies, including rabbit polyclonal anti‐4E‐BP1 (9452S, Cell Signaling Technology, Danvers, MA, USA, 1 : 2000 dilution), rabbit polyclonal anti‐p‐4E‐BP1 (2855S, Cell Signaling Technology, 1 : 2000 dilution), total OXPHOS Rodent WB Antibody cocktail (ab110413, Abcam, Cambridge, UK, 1 : 1000), rabbit GAPDH (5174, Cell Signaling Technology, 1 : 4000 dilution), and mouse β‐actin (A1978, Sigma‐Aldrich, 1 : 3000).

After washing with TBS buffer (Thermo Fisher Scientific) containing 0.1% Tween‐20, the membranes were incubated with an anti‐rabbit IgG HRP‐linked whole antibody donkey secondary antibody (GE Healthcare Biosciences, Piscataway, NJ, USA) or anti‐mouse IgG HRP‐linked whole antibody sheep secondary antibody (Cytiva). Protein detection was performed using Immobilon Forte Western HRP substrate (Merck Millipore, Darmstadt, Germany), and band intensity was quantified using the imagej software (version 1.53, National Institutes of Health, Bethesda, Maryland, USA). Phosphorylation of 4EB‐P1 was calculated as the ratio of p‐4E‐BP1/4E‐BP1 after normalization against GAPDH.

### Imaging data and statistical analysis

The X‐Y drift of the imaging data was corrected using the imagej plug‐in Stackreg, and the fluorescence intensity (FI) of each cell was measured using the metamorph software. For imaging, the average FI during the 3‐min period before administration was normalized to 100% for each individual cell. The average normalized FI was calculated for each experiment. Data are shown as mean ± standard deviation. We calculated the area under the curve (AUC) value of the FI after the administration of single cells and calculated the mean value per trial. Statistical analyses were performed using the Mann–Whitney *U* test for two‐group comparisons and the Kruskal–Wallis test followed by Dunn's *post hoc* test for multiple comparisons among three groups using graphpad prism6 (GraphPad Software, San Diego, CA, USA).

## Results

### Mitochondrial ATP concentrations are decreased by administration of glucose to hepatocytes under glucose‐deprived conditions

**Fig. 1:**
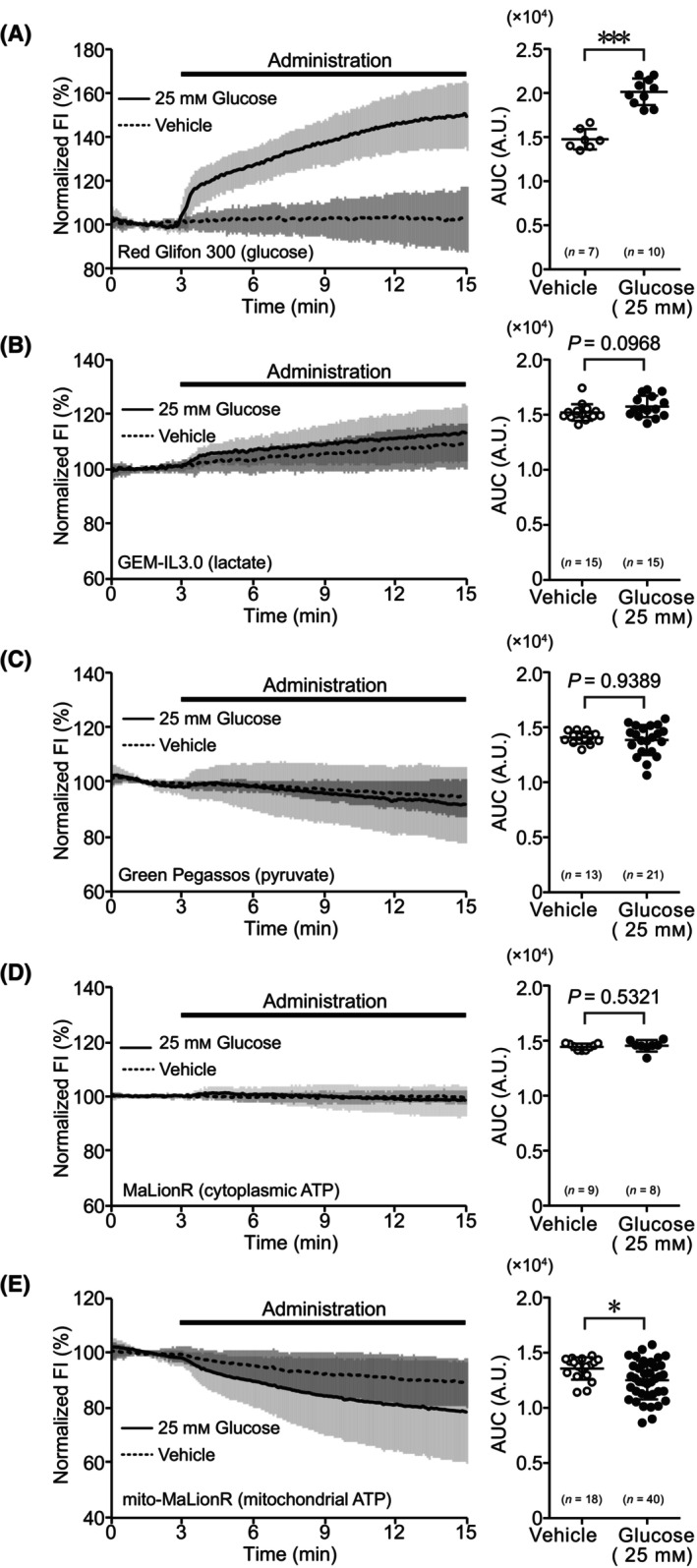
Dynamics of metabolism‐related molecules during administration of glucose to hepatocytes under glucose‐deprivation conditions. (A) Time course of fluorescence intensity (FI) and area under the curve (AUC) for Red Glifon 300. (B) Time courses of FI and AUC of GEM‐IL3.0. (C) Time course of FI and AUC for Green Pegassos. (D) Time course of FI and AUC for MaLionR. (E) Time course of FI and AUC for mito‐MaLionR. For time courses, the average of normalized FI in the data is shown as mean ± SD. FI during glucose administration is shown as a bold line, and the vehicle is shown as a dotted line. For AUC, data are shown as mean ± SD of values from independent experiments. Each dot represents AUC of the average value per trial. Statistical analyses were performed using the Mann–Whitney *U* test for two‐group comparisons. *P < 0.05, ***P < 0.001. *n*, number of trials.

We observed the dynamics of metabolites in hepatocytes derived from healthy mice under glucose‐deprivation/repletion conditions. Under glucose‐deprivation conditions, administration of 25 mm glucose to hepatocytes immediately increased the FI of Red Glifon 300, a sensor that is used to visualize intracellular glucose dynamics. The AUC of the time course of the FI was significantly higher than that under the unstimulated condition (Fig. 1A). Next, we confirmed whether administration of glucose under glucose‐deprivation conditions induced ATP production by the glycolytic system. In hepatocytes under glucose‐deprivation conditions, the FI of GEM‐IL3.0, which is used to visualize intracellular lactate dynamics, of Green Pegassos, which is used to visualize pyruvate dynamics, and of MaLionR, which is used to visualize cytoplasmic ATP dynamics, were not significantly different from that in unstimulated cells (Fig. 1B–D). To confirm whether administration of glucose induces ATP production via the TCA cycle and electron transfer system, we observed changes in the FI of mito‐MaLionR, which is used to visualize mitochondrial ATP dynamics. Under glucose‐deprivation conditions, administration of 25 mm glucose reduced the FI of mito‐MaLionR, and its AUC was significantly lower than that of unstimulated cells (Fig. 1E).

**Fig. 2:**
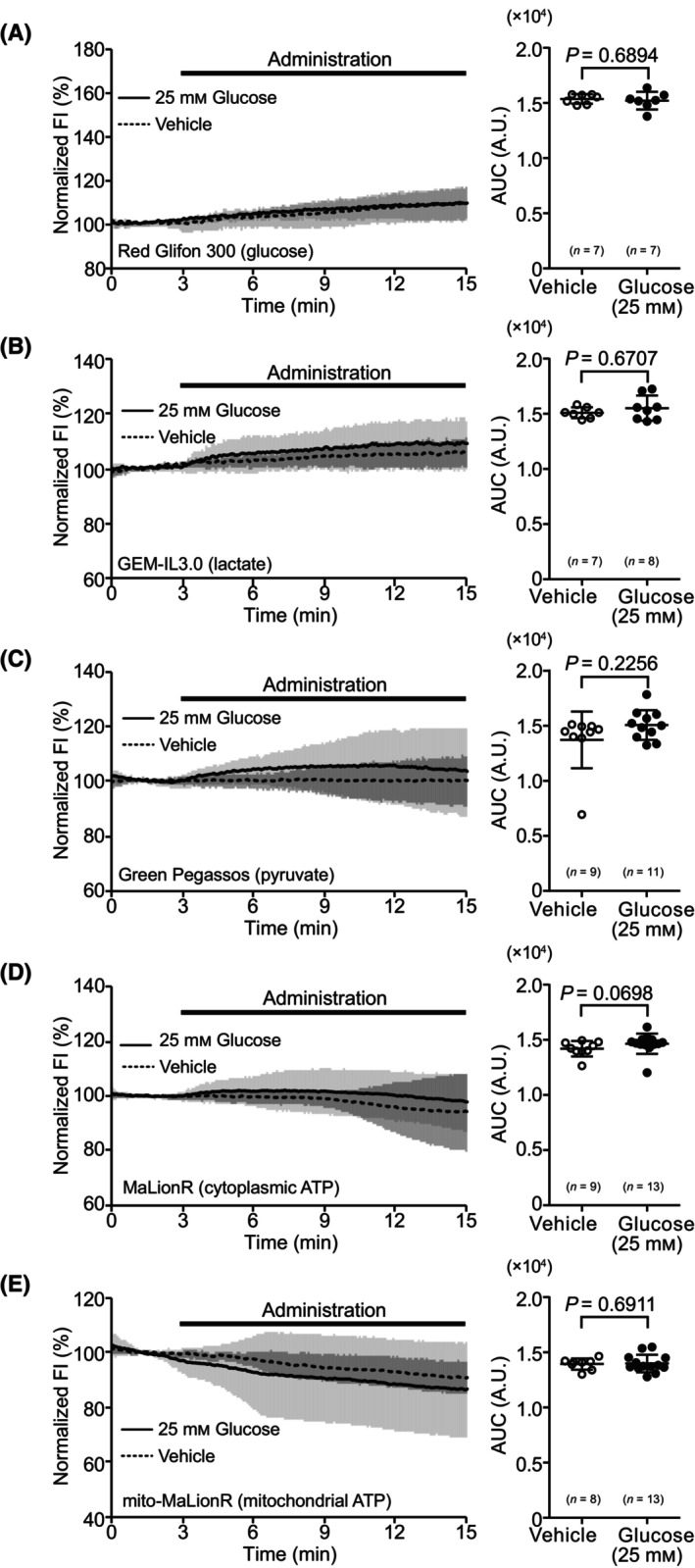
Dynamics of metabolism‐related molecules during administration of glucose to hepatocytes under glucose‐repletion conditions. (A) Time course of fluorescence intensity (FI) and area under the curve (AUC) for Red Glifon 300. (B) Time course of FI and AUC for GEM‐IL3.0. (C) Time course of FI and AUC for Green Pegassos. (D) Time course of FI and AUC for MaLionR. (E) Time course of FI and AUC for mito‐MaLionR. For time courses, the average of normalized FI in the data is shown as mean ± SD. FI during glucose administration is shown as a bold line, and the vehicle is shown as a dotted line. For AUC, data are shown as mean ± SD of values from independent experiments. Each dot represents AUC of the average value per trial. Statistical analyses were performed using the Mann–Whitney *U* test for two‐group comparisons. *n*, number of trials.

In contrast, under glucose‐repletion conditions, no sensor showed significant changes in FI upon the administration of 25 mm glucose, and its AUC was not significantly different from that under the unstimulated condition (Fig. 2).

**Fig. 3:**
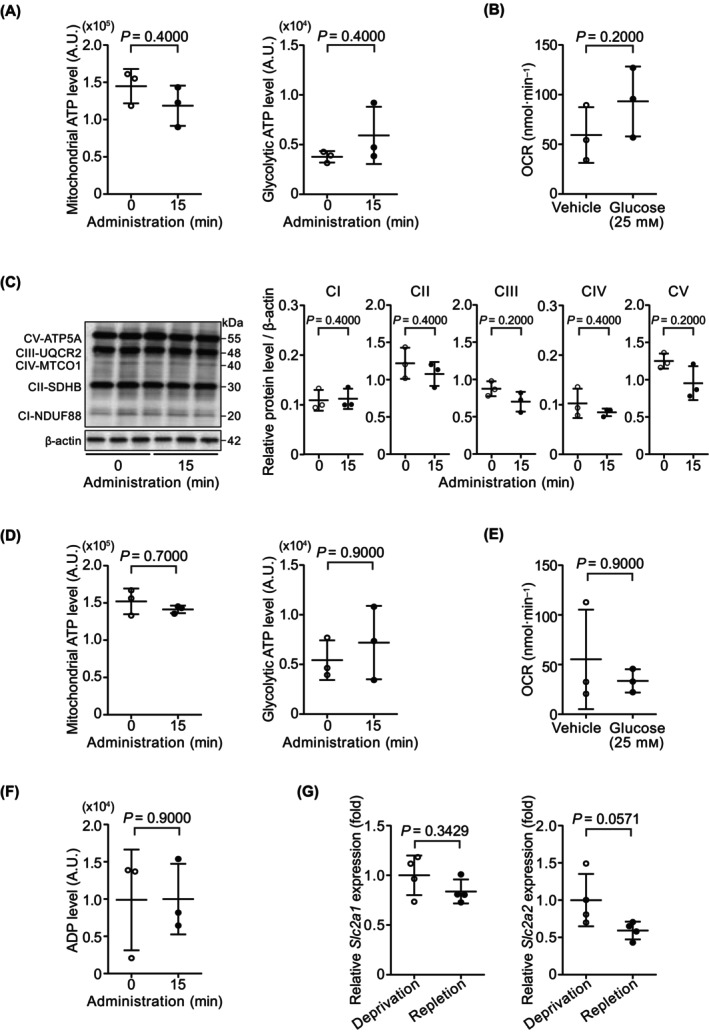
Status of glycolysis and oxidative phosphorylation during glucose administration. (A) Mitochondrial and glycolytic ATP levels assessed based on luminescence intensity (LI) after glucose administration under glucose‐deprivation conditions. Data are shown as mean ± SD of values from three independent experiments. (B) Oxygen consumption rate (OCR) after glucose administration under glucose‐deprivation conditions. Data are shown as mean ± SD of values from three independent experiments. (C) Western blot analysis of OXPHOS complex I‐V (CI‐CV) in hepatocytes under glucose‐deprivation conditions after glucose administration. CI‐CV were normalized against β‐actin. Data are presented as mean ± SD of values from three independent experiments. (D) Mitochondrial and glycolytic ATP levels assessed based on LI after glucose administration under glucose‐repletion conditions. Data are shown as mean ± SD of values from three independent experiments. (E) OCR after glucose administration under glucose‐repletion conditions. Data are shown as mean ± SD of values from three independent experiments. (F) ADP levels assessed using LI after glucose administration under glucose‐deprivation conditions. Data are shown as mean ± SD of values from three independent experiments. (G) Comparison of *Slc2a1* and *Slc2a2* expression in hepatocytes between glucose‐deprivation and glucose‐repletion conditions. Data are presented as mean ± SD of values from four independent experiments. Statistical analyses were performed using the Mann–Whitney *U* test for two‐group comparisons.

Next, we investigated whether oxidative phosphorylation (OXPHOS) is restricted by glucose administration under glucose‐deprived conditions. Under glucose‐deprivation conditions, mitochondrial and glycolytic ATP levels determined using biochemical analysis were only slightly changed after glucose administration (Fig. 3A). Oxygen consumption rate (OCR) was also changed only slightly after glucose administration (Fig. 3B). In addition, the expression of proteins related to OXPHOS was only slightly changed (Fig. 3C). Under glucose‐repletion conditions, administration of glucose did not alter mitochondrial and glycolytic ATP levels, or OCR (Fig. 3D,E). A decrease in mitochondrial ATP levels after glucose administration could also be caused by a decrease in total adenine nucleotides. We measured ADP levels, which were changed only slightly after glucose administration (Fig. 3F).

In addition, we confirmed the status of glucose transporter in hepatocytes under glucose depletion and repletion conditions. GLUT1 (SLC2A1) is widely expressed in different tissues including the liver, and GLUT2 (SLC2A2) is the major glucose transporter in the hepatocytes in rodents [20]. No significant differences were observed in the expression of *Glut1* under both glucose‐depletion and glucose‐repletion conditions (Fig. 3G left). On the contrary, the expression of *Glut2* under glucose‐depletion conditions tended to be lower than that under repletion conditions (Fig. 3G right).

### Decrease in mitochondrial ATP concentration with glucose administration is unlikely to be related to glycogen synthesis

**Fig. 4:**
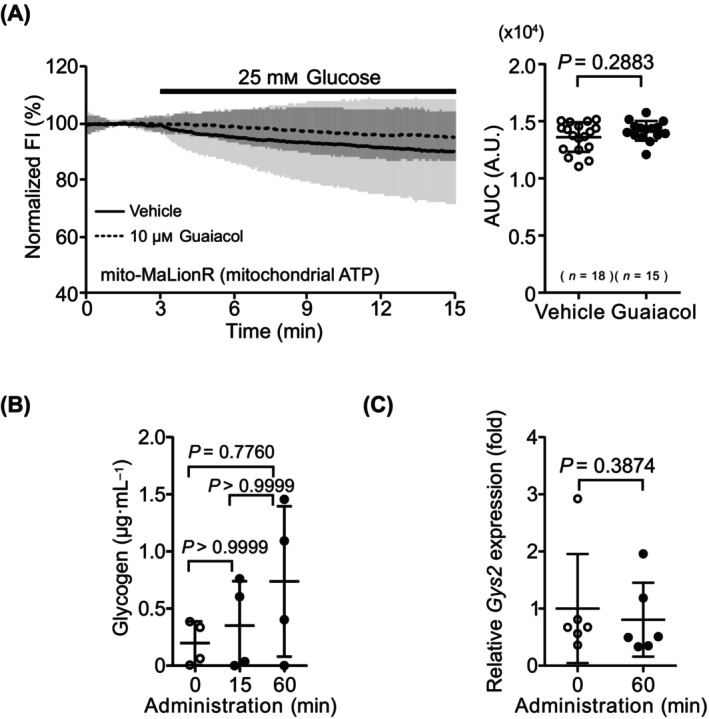
Involvement of glycogen synthesis in decreasing mitochondrial ATP concentration. (A) Time course of fluorescence intensity (FI) and area under the curve (AUC) of mito‐MaLionR during glucose administration after application of 10 μm guaiacol, a glycogen synthase inhibitor, under glucose‐deprivation conditions. For time course, the average of normalized FI in the data is shown as mean ± SD. FI during glucose administration in guaiacol untreated cells is shown as a bold line, and in guaiacol‐treated cells as a dotted line. For AUC, data are shown as mean ± SD of values from independent experiments. Each dot represents AUC of average per trial. *n*, number of trials. (B) Intracellular glycogen concentration after administration of glucose to deprived cells. Data are presented as mean ± SD of values from four independent experiments. (C) Comparison of *Gys2* expression in glucose‐deprived hepatocytes after glucose administration. Data are presented as mean ± SD of values from six independent experiments. Statistical analyses were performed using the Mann–Whitney *U* test for two‐group comparisons and the Kruskal–Wallis test followed by Dunn's *post hoc* test for multiple comparisons among three groups.

We investigated the factors involved in the decrease in mitochondrial ATP concentration caused by glucose administration under glucose‐deprivation conditions. First, we considered the possibility that hepatocytes use ATP produced in the mitochondria for glycogen synthesis, which is the main pathway for glucose utilization and is depleted during deprivation. Hepatocytes were treated with 10 μm guaiacol, a glycogen synthase inhibitor, for 1 h under glucose‐deprivation conditions, and the FI of mito‐MaLionR in hepatocytes was observed during the administration of 25 mm glucose. The AUC of the mito‐MaLionR FI in guaiacol‐treated cells showed little change compared with that in untreated cells (Fig. 4A). Next, we measured the intracellular glycogen concentration and found that it did not increase in hepatocytes under glucose‐deprivation conditions after glucose administration (Fig. 4B). We also compared the expression level of glycogen synthase *Gys2* and found only a slight change with or without glucose administration under glucose‐deprivation conditions (Fig. 4C). These data suggested that glycogen synthesis was not related to the decrease in mitochondrial ATP concentration following glucose administration.

### Decrease in mitochondrial ATP concentration with glucose administration may be involved in protein synthesis

**Fig. 5:**
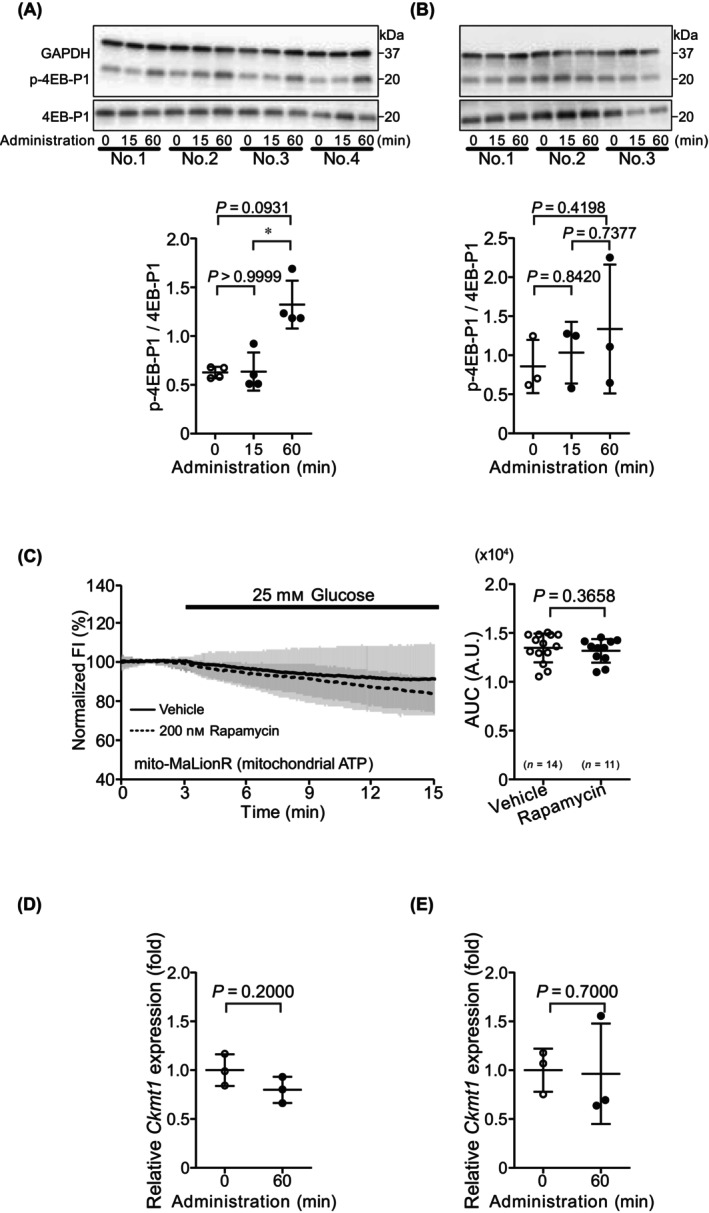
Involvement of translation in decreasing mitochondrial ATP concentration. (A) Western blots showing the expression of 4E‐BP1, phospho‐4E‐BP1, and GAPDH (loading control) for 1 h in hepatocytes under glucose‐deprivation conditions after glucose administration. Densitometric analysis of the phospho‐4EB‐P1/4E‐BP1 ratio. The expression of 4E‐BP1 and phospho‐4E‐BP1 was normalized against GAPDH. Data are presented as mean ± SD of values from four independent experiments. (B) Western blots showing the expression of 4E‐BP1, phospho‐4E‐BP1, and GAPDH (loading control) in hepatocytes under glucose‐repletion conditions after glucose administration. Densitometric analysis of the phospho‐4EB‐P1/4E‐BP1 ratio. The expression of 4E‐BP1 and phospho‐4E‐BP1 was normalized against GAPDH. Data are presented as mean ± SD of values from three independent experiments. (C) Time course of fluorescence intensity (FI) and area under the curve (AUC) of mito‐MaLionR during glucose administration after application of 200 nm rapamycin, an mTORC1 inhibitor, for 1 h under glucose‐deprivation conditions. For the time courses, the average of normalized FI is presented as mean ± SD. FI during glucose administration in non‐rapamycin‐treated cells is shown as a bold line and FI in rapamycin‐treated cells is shown as a dotted line. For AUC, data are presented as mean ± SD of values from independent experiments. Each dot represents AUC of average per trial. *n*, number of trials. (D) Comparison of *Ckmt1* expression in glucose‐deprived hepatocytes after glucose administration. Data are presented as mean ± SD of values from three independent experiments. (E) Comparison of *Ckmt1* expression in glucose‐repleted hepatocytes after glucose administration. Data are presented as mean ± SD of values from three independent experiments. Statistical analyses were performed using the Mann–Whitney *U* test for two‐group comparisons and the Kruskal–Wallis test followed by Dunn's *post hoc* test for multiple comparisons among three groups. *P < 0.05.

We investigated whether the decrease in mitochondrial ATP concentration is related to the synthesis of energy metabolism‐related proteins during the switching of the metabolic state from fasting to feeding. Translation is considered one of the most energy‐consuming processes in cells [21]. Phosphorylation of eukaryotic translation initiation factor 4E‐binding protein 1 (4E‐BP1), which is involved in the initiation of protein synthesis, was significantly increased after 60 min compared with that after 15 min of glucose‐deprivation (Fig. 5A). In contrast, 4EB‐P1 phosphorylation was not induced upon glucose administration under glucose‐repletion conditions (Fig. 5B). The mammalian target of rapamycin complex 1 (mTORC1) regulates 4E‐BP‐dependent translation [22]. Hepatocytes were treated with 200 nm rapamycin, an mTORC1 inhibitor, for 1 h under glucose‐deprivation conditions, and the mito‐MaLionR FI was observed in hepatocytes during then 25 mm glucose treatment. The AUC of the mito‐MaLionR FI in mTORC1‐inhibited hepatocytes was similar to that in untreated cells (Fig. 5C). These results suggest that glucose administration is likely to enhance translation after a decrease in mitochondrial ATP concentration.

Because protein synthesis was enhanced after glucose administration, we tested the possibility that mitochondrial ATP is transported to the cytoplasm. We quantified the expression level of mitochondrial creatine kinase (*Ckmt1*), which is related to the energy transport function between mitochondria and cytoplasm [23]. The expression of *Ckmt1* was only slightly changed after glucose administration under both glucose‐deprivation (Fig. 5D) and glucose‐repletion conditions (Fig. 5E).

### Decrease in mitochondrial ATP concentration with glucose administration is unlikely to be related to the activation of the urea cycle and reactive oxygen species

**Fig. 6:**
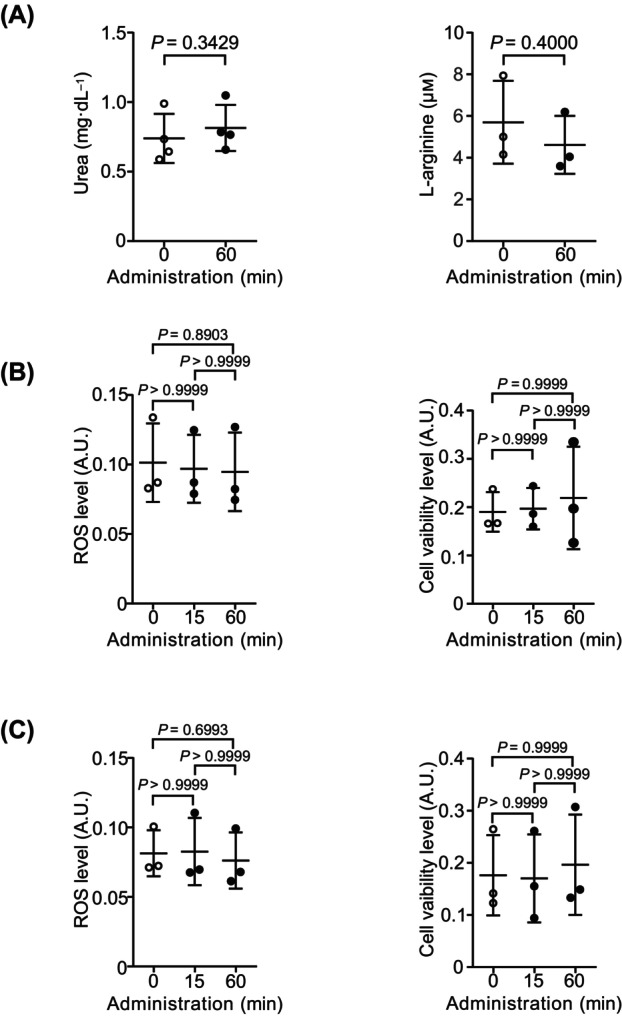
Involvement of the activation of urea cycle and reactive oxygen species (ROS) in decreasing mitochondrial ATP concentration. (A) Intracellular concentration of urea and l‐arginine after administration of glucose to deprived cells. Data are presented as mean ± SD of values from three independent experiments. (B) Intracellular ROS levels and cell viability assessed using fluorescence intensity (FI) after glucose administration under glucose‐deprivation conditions. Data are presented as mean ± SD of values from three independent experiments. (C) Intracellular ROS levels and cell viability assessed using FI after glucose administration under glucose‐repletion conditions. Data are presented as mean ± SD of values from three independent experiments. Statistical analyses were performed using the Mann–Whitney *U* test for two‐group comparisons and the Kruskal–Wallis test followed by Dunn's *post hoc* test for multiple comparisons among three groups.

Proteins are constantly degraded into their constituent amino acids for protein reconstitution. Amino acid turnover produces ammonia, which is subsequently detoxified to urea in the urea cycle. Urea biosynthesis requires ATP, which is less than 20% of the energy derived from gluconeogenic amino acid metabolism [24]. Because protein synthesis was increased after glucose administration, we measured the levels of urea/l‐arginine synthesized under glucose‐deprivation conditions. However, the intracellular urea (Fig. 6A left) and l‐arginine (Fig. 6A right) concentrations were only slightly changed after glucose administration.

Reactive oxygen species (ROS) are mainly generated by the mitochondrial electron transfer chain in hepatocytes [25]. We investigated whether administration of glucose produces excessive ROS and affects cell survival. Reactive oxygen species levels (Fig. 6B left) and cell viability (Fig. 6B right) in hepatocytes were only slightly changed after glucose administration under both glucose‐deprivation and glucose‐repletion conditions (Fig. 6C).

These results suggest that urea synthesis and ROS production are unlikely to be involved in the decrease in mitochondrial ATP after administration of glucose under glucose‐deprivation conditions.

### Effect of glucose administration to hepatocytes from obese model mice under glucose‐deprivation conditions on intracellular metabolite levels

**Fig. 7:**
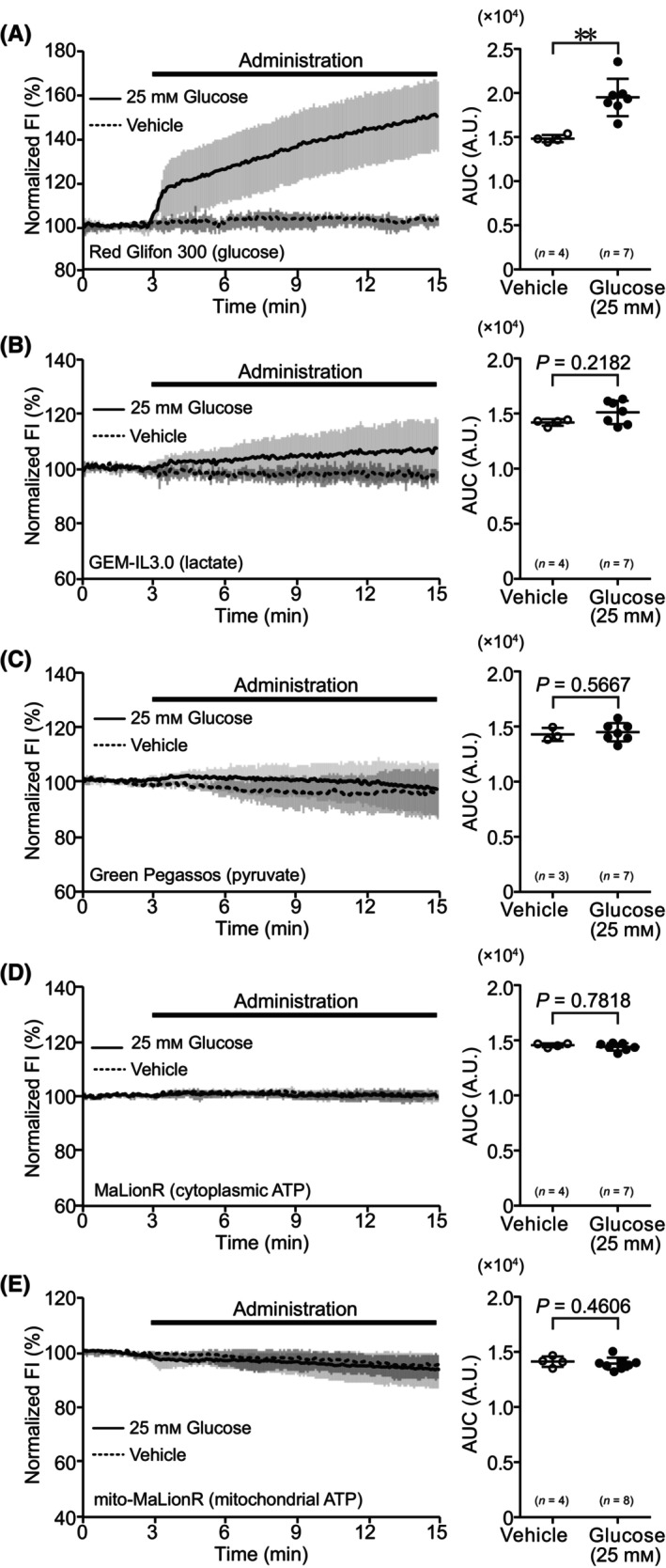
Dynamics of metabolism‐related molecules during administration of glucose to hepatocytes in an obese mouse model under glucose‐deprivation conditions. (A) Time course of fluorescence intensity (FI) and area under the curve (AUC) for Red Glifon 300. (B) Time course of FI and AUC for GEM‐IL3.0. (C) Time course of FI and AUC for Green Pegassos. (D) Time course of FI and AUC for MaLionR. (E) Time course of FI and AUC for mito‐MaLionR. For the time courses, the average of normalized FI in the data is presented as mean ± SD. FI during glucose administration is shown as a bold red line, and that during the vehicle administration is shown as a dotted line. For AUC, data are presented as mean ± SD of values from independent experiments. Each dot represents the AUC of the average for each trial. Statistical analyses were performed using the Mann–Whitney *U* test for two‐group comparisons. **P < 0.01. *n*, number of trials.

We examined the dynamics of metabolites in the hepatocytes derived from the obese mouse model. Under glucose‐deprivation conditions, the administration of 25 mm glucose to hepatocytes immediately increased the FI of Red Glifon 300, and the AUC was significantly higher than that under the unstimulated condition (Fig. 7A). The FI of GEM‐IL3.0, Green Pegassos, and MaLionR was not significantly different from that of the unstimulated cells after glucose administration (Fig. 7B–D). These responses were similar to those observed in hepatocytes from healthy mice. In contrast, glucose administration caused only a slight change in the FI of mito‐MaLionR, a result that was different from that obtained in hepatocytes from healthy mice (Fig. 7E).

## Discussion

In this study, we investigated the metabolic dynamics of primary hepatocytes under glucose deprivation/repletion conditions immediately after glucose administration. Live‐cell imaging analysis at the single‐cell level revealed that administration of glucose to hepatocytes under glucose‐deprivation conditions can cause an immediate decrease in the mitochondrial ATP concentration. In contrast, cytoplasmic ATP concentration showed little change.

Based on the measurement of glycolysis, OXPHOS, and OCR, it is likely that the amount of ATP produced in the cytoplasm and mitochondria was only slightly changed after glucose administration. Therefore, we thought that after glucose administration, ATP is not used to increase energy production, but to restart other functions that were suppressed during glucose depletion.

Administration of glucose differentially affected mitochondrial and cytosolic ATP concentrations. Increased ATP consumption in the cytoplasm is compensated by ATP supply from mitochondria [26, 27]. ATP supply from mitochondria may occur for protein synthesis. In muscle, the mitochondrial creatine kinase isoform is known to be involved in energy transfer to the cytosol and in the stimulation of oxidative phosphorylation [23, 28]. However, the expression of *Ckmt1* is normally low in the liver [29]. Indeed, there was no significant difference in the presence or absence of glucose administration in the present study. In the future, it would be necessary to verify whether ATP is transported from mitochondria to the cytosol via other pathways, such as through the ATP‐Mg/Pi carrier [30].

GLUT1 is expressed in various organs and is thought to be responsible for basal glucose uptake. GLUT2 is the major glucose transporter in hepatocytes of rodents and humans, and is a bidirectional transporter, taking up glucose during the absorptive phase and releasing it into the blood during fasting [20]. No significant differences in their expression levels between the glucose‐depletion and glucose‐repletion conditions were noted in this study. Translocation of GLUT1 and GLUT2 to the membrane in both the models was not confirmed and requires further work to understand whether the models reflect the responses *in vivo*.

In hepatocytes, the major pathway of glucose utilization is glycogen synthesis, and approximately 50% of ingested glucose is stored as glycogen during the postprandial period in healthy individuals [31]. During fasting, hepatic glycogen concentration decreases, and glycogen is synthesized within a few minutes upon refeeding [32, 33]. Exogenous glucose is converted to glucose‐6‐phosphatase and glycogen is synthesized via UDP‐glucose. The UTP consumed in this reaction is synthesized from UMP and UDP using ATP. Previous experiments with isolated perfused livers from rats fed after 48 h of fasting suggested that mitochondrial ATP may be related to UTP synthesis, which is required for the initial phase of glycogen synthesis [34]. However, the present study suggests that intracellular glycogen synthesis is unlikely to occur immediately following the glucose administration. These results suggest that the decrease in the mitochondrial ATP concentration is related to other basal cellular activities.

Gallis et al. [34] showed that the metabolic turnover of mitochondrial ATP during glycogen synthesis is equivalent to ~ 50% of the basal flux of mitochondrial ATP production, suggesting that the remaining 50% may maintain basal cellular activities. Translation is an elongation step in which new amino acids are added to a growing polypeptide chain by consuming at least two molecules of ATP and GTP, which is an energetically costly process. Therefore, cells are equipped with several regulatory mechanisms to ensure that translation occurs only in the presence of sufficient nutrients and growth factors [35]. One of these is AMP‐activated protein kinase (AMPK), which is an intracellular sensor of energy and nutrients. Deprivation activates AMPK and blocks the mTORC1 signaling pathway. As a result, mTORC1 dephosphorylates 4E‐BP1 to halt translation [22]. Translation has recently been shown to be halted by glucose deprivation even when amino acids are adequately supplied [36]. We tested the possibility that glucose administration could initiate translation that had been halted under glucose‐deprivation conditions, resulting in the use of ATP produced in the mitochondria. Imaging for 15 min did not reveal a relationship between the decrease in mitochondrial ATP concentration and protein synthesis. As long‐term imaging is difficult owing to limitations, such as sensor photobleaching, improved techniques should be investigated. Rapamycin is widely used as an inhibitor of mTORC1; however, a recent study reported that it may not inhibit cap‐dependent translation initiation [37]. In the future, it will be necessary to verify the effect of inhibition by other methods on the decrease in the mitochondrial ATP concentration.

Hepatic mitochondria are nutrient sensors, and their biosynthesis and dynamics are regulated to adapt to hepatic metabolism [38, 39]. In this study, the reduction in mitochondrial ATP concentration due to glucose‐repletion was found to be different in obese and normal mice. Although the physiological significance of this phenomenon remains to be elucidated, it may be related to abnormal energy metabolism in obesity. Mitochondria under overnutrition increase the β‐oxidation rate, enzyme activity of the mitochondrial electron transfer system, and ketogenesis to adapt to hepatic energy substrate overload. In fact, these increases have been reported in obese subjects without NAFLD and in patients with early‐stage NAFLD compared with healthy controls [40]. Analysis using metabolic disorder, such as NAFLD, nonalcoholic steatohepatitis (NASH), and type 2 diabetes models, may help visualize mitochondrial function during progression to NASH and type 2 diabetes. Intermittent fasting and time‐restricted eating have attracted attention as a dietary approach for NAFLD [41]. These have been reported to improve metabolism, including weight loss and improvement of insulin resistance, not only in rodents but also in humans [41, 42, 43]. Our results partially reveal the mechanism underlying the adaptation to nutrient deprivation and repletion in hepatocytes. Further molecular and signaling analysis of the effects of nutrient depletion and subsequent repletion at the cellular and tissue level may help in developing therapeutic strategies. In addition, refeeding might primarily affect lipid metabolism in the liver, according to findings in a study wherein gene expression induced in the liver by fasting and refeeding was comprehensively analyzed [5, 8]. To further investigate the metabolic switch, lipid and ketone metabolism should be visualized immediately after glucose refeeding. Other responses known to be affected by fasting and feeding include iron metabolism, endoplasmic reticulum stress responses, and hepatic autophagy [6]. Future studies should analyze the relationship between these factors and mitochondrial ATP levels in detail.

The present study has several limitations. First, deprivation and administration were mimicked only with glucose; therefore, the model differed from the fasting and feeding environments *in vivo*. Second, the primary culture conditions did not interact with other organs and did not reproduce hormone secretion or fuel supply from other organs. Third, in this experiment, glucose‐deprivation and administration were performed several days after collection from the living body to ensure a period of expression for each indicator. Fourth, a pattern of different metabolic capacities depending on the location, known as liver zonation of the hepatic lobule [44], was not considered in primary hepatocytes.

In the future, *in vivo* imaging should be performed using individuals with fluorescent protein indicators of metabolic molecules expressed in the liver.

## Conflict of interest

The authors declare no conflict of interest.

## Author contributions

ST, KH, MiM, TK, MYH, MaM, and TT conceived and designed the experiments. ST and MH performed the experiments. ST, KH, TK, MYH, MaM, and TT analyzed the data and wrote the manuscript. All authors reviewed and approved the final manuscript.

## Data Availability

The data that support the findings of this study are available from the corresponding author (takatsuboi@bio.c.u-tokyo.ac.jp) upon reasonable request.
